# Multimorbidity profile among cancer-related hospitalization events in younger and older patients: a large-scale nationwide cross-sectional study

**DOI:** 10.1016/j.lana.2025.101308

**Published:** 2025-11-25

**Authors:** Yanara A. Bernal, Carla Campaña, Cristobal Sanhueza, Mauricio Apablaza, Ricardo Armisén, Iris Delgado

**Affiliations:** a School of Nursing, Pontificia Universidad Católica de Chile, Santiago, Chile; b Center of Global Intercultural Health (CeSGI), Facultad de Medicina Clínica Alemana Universidad del Desarrollo, Facultad de Psicología Universidad del Desarrollo, Santiago, Chile; c Center for Cancer Prevention and Control (CECAN), FONDAP 152220002 ANID, Chile; d Departamento de Oncología, Facultad de Medicina Clínica Alemana, Universidad del Desarrollo, Santiago, 7550000, Chile; e Facultad de Gobierno, Universidad del Desarrollo, Av. Plaza #680, San Carlos de Apoquindo, 7610658, Las Condes, Santiago, Chile; f Centro de Genética y Genómica, Instituto de Ciencias e Innovación en Medicina, Facultad de Medicina Clínica Alemana, Universidad del Desarrollo, Santiago, 7550000, Chile; g Centro de Epidemiología y Políticas de Salud, Instituto de Ciencias e Innovación en Medicina, Facultad de Medicina Clínica Alemana, Universidad del Desarrollo, Santiago, 7550000, Chile

**Keywords:** (MeSH) (3–10): multimorbidity, Chronic disease, Cancer, Hospitalization, Early cancer, Diagnosis-related groups, Obesity, Diabetes, Hypertension

## Abstract

**Background:**

Multimorbidity, the coexistence of two or more chronic diseases, among cancer patients offers critical insights into shared risk factors, while posing increasing challenges for healthcare systems due to the complexity of care required. Despite its relevance, research in multimorbidity across different age groups is limited in middle income countries.

**Methods:**

We analyzed cancer-related hospitalizations between 2019 and 2023, using a nationwide Diagnosis-Related Groups database covering 68 Chilean health institutions. We examined the distribution of 40 chronic conditions, multimorbidity prevalence, comorbidity profile, and their distribution across age group, sex, and cancer diagnosis.

**Findings:**

We identified 4,722,723 hospitalization events, including 149,270 unique adult patients hospitalized with cancer (mean of 63 ± 15.17 years old). Multimorbidity was present in 47.9% of all cancer-related hospitalizations, increasing steeply with age: 14% in patients aged 18–35, 24.9% in those 36–50, and 55.5% in patients >50 years. Obesity and diabetes were among the most common comorbid conditions across age groups, with significant variations by sex. Notably, obesity was more prevalent in younger patients, particularly those aged 18–35, whereas hypertension showed an inverse profile, increasing markedly with age.

**Interpretation:**

Multimorbidity profile reflect both the clinical complexity of cancer care and potential shared biological and environmental pathways in carcinogenesis. These findings highlight the need to transition from disease-centered to person-centered care models. In Chile, understanding multimorbidity in younger and middle-aged adults may inform precision prevention, integrated service delivery, and equitable planning for both oncologic and non-oncologic care.

**Funding:**

This study was conducted without external funding.

**Research in context**

**Evidence before this study**

Multimorbidity in cancer patients is associated with higher rates of hospitalization, complications, and mortality, posing a growing challenge for health systems. However, most of the existing evidence comes from high-income countries, particularly in Europe and North America, where electronic health records and national cancer registries are available. We conducted a search in PubMed and regional health databases for studies published between January 2010 and January 2025, using the terms “multimorbidity AND cancer,” “comorbidities AND hospitalization AND Latin America,” and “chronic disease AND oncology.” The search identified hundreds of studies addressing multimorbidity, including dozens conducted in Chile; however, none specifically focused on multimorbidity among individuals with cancer. Available evidence in the region remains scarce and fragmented. Moreover, comparisons between younger and older cancer patients are limited, despite the likelihood of significant differences in multimorbidity profiles across age groups. To our knowledge, no previous study has used high-quality national hospital data to characterize multimorbidity profile in cancer patients in Chile. This study aims to fill that gap by providing a large-scale analysis of cancer-related hospitalizations and associated chronic conditions in the Chilean population.

**Added value of this study**

This is the first study to analyze over 4.7 million hospitalizations from a nationwide Chilean Diagnosis-Related Groups (DRG) database, identifying nearly 150,000 adult cancer patients and evaluating 40 chronic conditions. We provide a detailed description of multimorbidity prevalence and combinations by age group, cancer type, and sex. The results reveal pronounced age gradients, with distinct multimorbidity profile in younger vs. older adults. Obesity and anemia were more prevalent among younger patients, while hypertension and diabetes predominated in older adults. These profiles also varied by cancer type and sex, underscoring the clinical and epidemiological complexity of multimorbidity.

**Implications of all the available evidence**

Understanding age- and sex-related differences in multimorbidity among cancer patients is essential for designing clinical interventions and health policies tailored to real-world population needs. Our findings emphasize the urgency of shifting from disease-centered approaches to integrated, person-centered care models. This evidence is particularly relevant to health systems in Latin America, where high chronic disease burden coexists with increasing cancer survivorship. This study provides essential data to guide precision prevention, promote health equity, and strengthen the planning of multidisciplinary care services for cancer patients with multimorbidity across different age groups.

## Introduction

Advances in cancer detection, diagnosis, and treatment have improved cancer survival worldwide, creating a growing population of individuals living with or beyond cancer.^1^ This demographic shift presents new and complex challenges for health systems, particularly in low- and middle-income countries, where resources for long-term survivorship care remain limited.^2^ Over 28 million new cancer cases are projected annually by 2040, with the largest increases expected in regions such as Latin America.^3^ In this context, the growing survivorship demands a better understanding of the burden of chronic comorbidities, especially in regions with scarce longitudinal evidence.

Cancer survivors face elevated risks of late treatment effects, accelerated biological aging, and the development of chronic comorbidities, including cardiovascular disease, diabetes, and renal dysfunction.^4^ These consequences not only compromise quality of life but also increase healthcare utilization and strain public health infrastructure. Currently, the overall 5-year relative survival rate for all cancers combined is now approximately 68%.^4^ As the number of cancer survivors grows, so too does the imperative to understand and address the long-term burden of multimorbidity in this population, including how chronic conditions co-occur across different patient groups to guide clinical and public health strategies.

Multimorbidity, defined as the coexistence of two or more chronic diseases in a subject,^5^ has become an increasingly common health issue worldwide. However, in this study, we applied a more restrictive operational definition, two or more chronic diseases in addition to cancer, to focus specifically on the burden of non-cancer comorbidities in cancer-related hospitalizations. This approach was chosen because cancer was the index condition of interest and including it in the multimorbidity count would overestimate the prevalence in our target population. To better capture its complexity, researchers have identified approximately forty chronic conditions that are frequently used in clinical and epidemiological studies based on electronic health records.^6^, ^7^, ^8^, ^9^ In Chile, evidence suggests that multimorbidity in older adults often clusters into distinct profiles, mechanical, cardio-metabolic, psycho-geriatric, affective-respiratory, and conditions such as colorectal polyps and benign prostatic hyperplasia,^10^ and has been associated with an increased risk of mortality.^11^ Over recent decades, this phenomenon has gained prominence due to rising life expectancy and the growing burden of chronic diseases,^12^ which together contribute to higher mortality rates and reduced quality of life in affected individuals.^13^ As its prevalence continues to rise, this phenomenon has emerged as one of the most pressing challenges for global public health,^14^ leading to increased healthcare demands, higher costs, and greater difficulties in managing polypharmacy.^15^ These challenges are particularly pronounced among individuals with cancer, who often experience a unique and more severe burden of coexisting chronic conditions.

Several studies have shown that multimorbidity is significantly more common among cancer survivors compared to individuals without a cancer.^15^^,^^16^ While this phenomenon has been extensively documented using electronic health records in European and North American countries, comparative analyses remain scarce in Latin America. Nonetheless, national prevalence estimates have been reported, including 17.5% in Colombia, 37.3% in Jamaica, 14.4% in Mexico, 15.5% in El Salvador, 16.8% in Brazil, and 18.3% in Panama.^17^ The presence of multimorbidity in cancer patients not only compromises quality of life but has also been identified as a strong predictor of 6- and 12-month mortality in colorectal^18^ and lung^19^ cancer among Spanish populations, as well as in any cancer type within Canadian cohorts.^20^ Given the lack of regional evidence, it is essential to characterize multimorbidity among cancer patients in Latin American countries such as Chile, using national health data to inform public policies and clinical decision-making grounded in local evidence.

This study aimed to analyze comorbidities and profile of multimorbidity among cancer-related hospitalizations by age group in Chilean healthcare institutions between 2019 and 2023, using a large national hospital discharge database based on Diagnosis-Related Groups (DRGs) a patient classification system that groups hospital cases with similar clinical characteristics and expected resource use for the purposes of financing and performance evaluation. We examined the sociodemographic characteristics of hospitalized patients by age and multimorbidity status, in-hospital mortality, and identified the most prevalent chronic conditions across age groups and cancer types. Understanding these differences is crucial to tailoring healthcare interventions to the specific needs of patients with multiple chronic conditions, particularly younger and adult individuals with cancer. These distinctions underscore the importance of personalized and multidisciplinary approaches to managing cancer-related multimorbidity, with attention to the specific social and epidemiological context of Latin American populations.

## Methods

### Nationwide database

The data for this study were obtained from the Diagnosis-Related Groups (DRGs) database, which compiles 100% of hospitalization records from all Chilean hospitals that provide care to patients covered by the public health insurance system (FONASA). FONASA insures approximately 80% of the Chilean population, thereby ensuring broad representativeness of the national health system. These institutions include both high- and low-complexity facilities, offering a broad representation of the national health system. This database contains diagnoses based on the International Classification of Diseases, Tenth Revision (ICD-10) codes, which were used to identify cancer-related hospitalizations and chronic disease diagnoses relevant to multimorbidity analyses. Additionally, we used the discharge type variable (death vs. alive (Home discharge or home hospitalization, referral to other healthcare facilities, voluntary discharge, and unauthorized discharge; detailed in Table S2) to study in-hospital mortality. Potential biases include underreporting of chronic conditions managed in outpatient care, and possible misclassification of diagnoses due to administrative coding. The database was downloaded from the Chilean National Health Fund (Fondo Nacional de Salud, FONASA) on January 29, 2025. This cross-sectional observational study is reported in accordance with the STROBE (Strengthening the Reporting of Observational Studies in Epidemiology) guidelines.

### Cancer-related hospitalization events by age group

We classified the age of cancer-related hospitalization when the age of cancer-related hospitalization in younger patients was less or equal to 50 (18–34 and 35–50 years old) and older patients (over 50 years old) calculated as the difference between the admission date and the date of birth. We determined the reason of hospitalization was cancer, when the first diagnosis registered with any cancer. We included all adult (≥18 years) patients with a primary diagnosis of cancer; records with missing age or sex were excluded. The study included the entire eligible population; no sample size calculation was performed. In this database the first diagnosis is the reason of hospitalization base on ICD-10. Malignant neoplasms were classified as malignant neoplasms of lip, oral cavity and pharynx (C00–C14), malignant neoplasms of digestive organs (C15–C26), malignant neoplasms of respiratory and intrathoracic organs (C27–C39), malignant neoplasms of bone and articular cartilage (C40–C41), malignant neoplasms of skin (C43, C44), malignant neoplasms of mesothelial and soft tissue (C45–C49), malignant neoplasm of breast (C50), malignant neoplasms of female genital organs (C51–C58), malignant neoplasms of male genital organs (C60–C63), malignant neoplasms of urinary tract (C64–C68), malignant neoplasms of eye, brain and other parts of central nervous system (C69–C72), malignant neoplasms of thyroid and other endocrine glands (C73–C75), malignant neoplasms, stated or presumed to be primary, of lymphoid, hematopoietic and related tissue (C81–C95) were excluded in situ (D00–D09), and benign (D10–D36) neoplasm, and malignant neoplasms of ill-defined, other secondary and unspecified sites (C68, C76–C80 and C96).

### Chronic diseases classification

Each diagnostic group was defined by specific ICD-10 codes representing conditions such as high blood pressure (I10, I11, I12, I13, I14, I15), myocardial infarction (I20, I21, I25), other heart diseases (I30–I52), varicose veins (I83, I87), osteoarthritis (M15–M19), osteoporosis (M80–M82), arthritis (M05, M06, M79), chronic pain (M40–M54), chronic allergy (J30, L23–L29, K27–K29), asthma (J45), chronic bronchitis, emphysema, chronic obstructive pulmonary disease (COPD) (J40–J44), diabetes (E10–E14), stomach or duodenal ulcer (K25, K26), urinary incontinence or urine control problems (N39), high cholesterol (E78), vision problems (H17–H54), chronic skin problems (L20–L40), liver diseases (K70–K76), mental health conditions (F32, F33, F41, F99), stroke (I60–I69, G45), migraine or frequent headache (G43, R51), hemorrhoids (I84), thyroid problems (E00–E07), kidney problems (N00–N20), prostate problems in men (N40–N42), menopausal problems in women (N95), permanent injuries or defects caused by accidents (T90–T98), obesity (E66), loss of hearing (H90, H91), gallbladder diseases (K80, K81), atherosclerosis (I65–I73), diverticulosis (K57), neuropathies (G50–G64), dizziness (H81, H82, R42), dementia (F00–F05, G30–G31, R54), urinary incontinence (N39, R32), anemia (D50–D64), sexual disorders (F52, N48), insomnia (G47, F51), tobacco use disorders (F17), and gout (E79, M10) (Table S1). The selection of these conditions was informed by the framework proposed by Koller et al.,^7^ with minor adaptations to reflect the structure and availability of diagnoses in our dataset.

### Chronic diseases and multimorbidity profiles

A subject was considered to have multimorbidity when they have two or more chronic pathologies in addition to their acute or chronic health condition.^21^ In our study, “multimorbidity profile” refers to the distribution and frequency of chronic conditions across age groups and cancer types, rather than the application of formal statistical pattern recognition methods. We consider cancer as a chronic health condition, and we classify the presence of multimorbidity when the number of additional chronic diseases to this condition exceeds two out of forty. While if the subject had cancer and only one chronic disease was reported, it was not classified as multimorbidity. This more restrictive definition was selected to focus on the additional chronic disease burden beyond cancer in hospitalized patients, and reflects the structure and variables available in the DRG dataset.

### Statistical analysis

Available clinical and sociodemographic data were summarized using descriptive statistics, including measures of central tendency (mean) and dispersion (standard deviation, SD) for continuous variables such as age at hospitalization. Categorical variables, including sex, cancer type, nationality, and insurance coverage, were reported as frequencies and percentages. Records with missing values in key variables were excluded from the analysis; no imputation procedures were applied. Notably, the source database contains a very low proportion of missing data. Comparisons between age groups were conducted using the ANOVA test for continuous variables and Fisher's exact test or chi-square test for categorical variables, as appropriate. A p-value <0.05 was considered statistically significant. To evaluate factors independently associated with in-hospital mortality, we fitted a multivariable logistic regression model including sex, age group, and number of comorbidities as predictors. Crude mortality rates were first compared using chi-square and spearman tests, and adjusted odds ratios (ORs) with 95% confidence intervals (95% CI) were then estimated from the logistic regression. All analyses and visualizations were performed using R (R Studio Version 2023.06.1 + 524). This study used administrative data from a national health database that captures the majority of cancer-related hospitalizations in Chile. Given the population-level nature of the data, statistical inference was not required to estimate general population parameters, and observed differences are interpreted descriptively rather than inferentially.

### Ethics statement

This study used anonymized, publicly available data from the FONASA -DRG database, which contains no identifiable or individual-level personal information. All analyses were conducted using aggregated, non-identifiable records. The study protocol was reviewed by the *Scientific Ethics Committee of the Institute of Science and Innovation in Medicine (ICIM), Faculty of Medicine, Universidad del Desarrollo, Clínica Alemana*, which determined that the use of these de-identified public data did not require formal ethics approval and therefore granted an exemption.

## Results

### Selection and characterization of hospitalization events and subjects

We analyzed hospitalization records from 2019 to 2023, identifying a total of 4,772,723 hospitalization events across all years. The annual distribution was as follows: 1,151,475 events in 2019; 781,912 in 2020; 816,909 in 2021; 932,840 in 2022; and 1,039,587 in 2023. These fluctuations reflect the hospital activity during the study period, potentially influenced by external factors such as the COVID-19 pandemic. Since the unit of analysis was the individual patient, we identified subjects based on their unique ID. In total, we included 149,270 unique adult patients (aged 18 years and older) who were hospitalized with a primary diagnosis of cancer during the study period.

### Variation in sex and cancer diagnostic profiles across age groups among hospitalized cancer patients

**Fig. 1:**
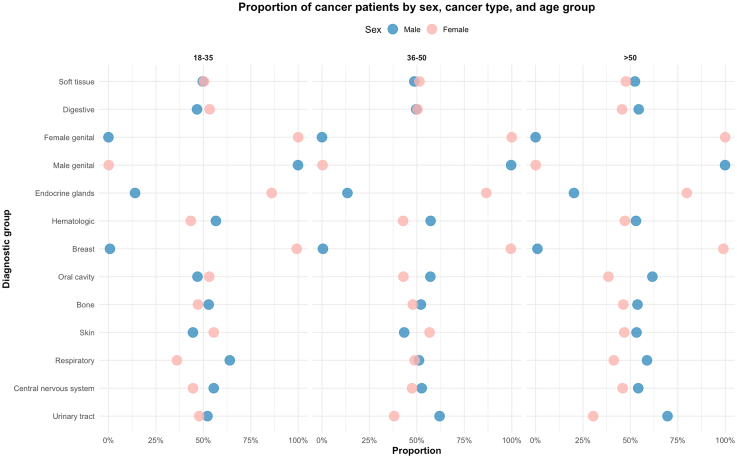
**Proportion of cancer patients by sex, cancer type, and age group**. Dot plot showing the proportion of male (blue) and female (pink) patients by cancer diagnostic group across three age categories: 18–35, 36–50, and >50 years identified during cancer-related hospitalizations in Chile between 2019 and 2023. Each point represents the relative proportion of one sex within a given cancer type and age group.

Fig. 1 shows the proportion of sex differences by cancer type and age group. Notable variations in sex distribution were observed across diagnostic categories and age subgroups, particularly in cancers of the oral cavity and urinary tract, where the proportion of female patients increased with age in the former and decreased in the latter. Additionally, in Table S2 shows the sociodemographic characteristics of the study population stratified by age group. Also, the average number of hospitalizations among cancer patients varied by age group. Individuals aged 18–35 had the highest mean number of hospitalizations (mean = 1.41, SD = 0.96), followed by those aged 36–50 (mean = 1.33, SD = 0.76), and adult patients showed the lowest average (mean = 1.26, SD = 0.62). These profiles suggests that younger adults with cancer may experience a higher frequency of hospital admissions, potentially reflecting differences in treatment intensity, follow-up needs, or cancer types prevalent in this age group.

### Cancer type frequency by age group

**Fig. 2:**
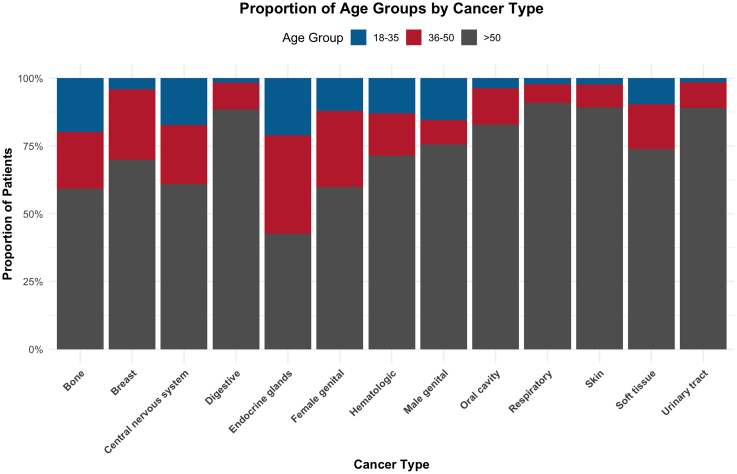
**Proportion of age groups by cancer type**. Proportion of patients stratified by age group (18–35, 36–50, and >50 years) across cancer types identified during cancer-related hospitalizations in Chile between 2019 and 2023.

When stratifying by age, distinct cancer prevalence profile emerged (Fig. 2). Fig. 2 present the proportion of patients in each age group across cancer types. In the text, we also report absolute counts (n) for each cancer type within age strata to provide phenomenon magnitude on the underlying group sizes. Among individuals aged 18–35 years (n = 9628), the most common cancer types were female genital (17.18%, n = 1654), and male genital cancers (16.77%, n = 1615), glandular tumors (16.69%, n = 1598), hematologic malignancies (15.76%, n = 1557), followed by breast cancer (10.48%, n = 1009). In the 36 to 50 age group (n = 24,031), breast cancer predominated (26.79%, n = 6438), followed by digestive (19%, n = 4567) and female genital (16.1%, n = 3869). For patients aged over 50 years (n = 115,610), the most prevalent cancer types were digestive (35.65%, n = 41,211), breast (14.95%, n = 17,289), urinary (9.24%, n = 10,688) and respiratory (6.6%, n = 7634). These differences highlight age-related oncologic profiles, with hematologic and central nervous system cancers more frequent among younger adults, and digestive, respiratory, and urinary tract cancers predominant in older populations.

### Multimorbidity prevalence by cancer type

**Fig. 3:**
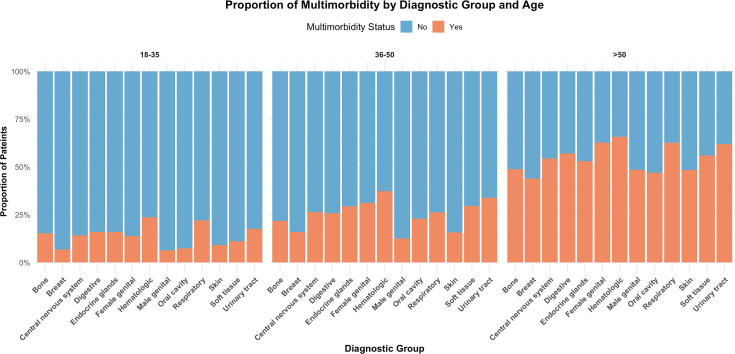
**Proportion of multimorbidity by diagnostic group and age**. Distribution of multimorbidity status (presence of ≥2 chronic conditions) by cancer type and age group.

Multimorbidity was present in 14% of patients aged 18–35 (n = 1353/9628), 24.9% in those aged 36–50 (n = 5980/24,031), and 55.5% in patients over 50 (n = 64,196/115,610), highlighting a steep age-related gradient in chronic disease burden among cancer-related hospitalizations. The prevalence of multimorbidity among cancer patients varied substantially across diagnostic groups and age categories (Fig. 3). Fig. 3 shows the proportion of patients with multimorbidity across cancer types by age group. We complement these proportions with absolute counts and denominators (n/N) to provide context on the underlying group sizes for each cancer type within age strata. In the 18–35 age group, the overall burden of multimorbidity was relatively low but not negligible; the highest proportions were observed in hematologic (23.6%), respiratory (22%), and urinary tract cancers (17.6%). Among adults aged 36–50, multimorbidity was more frequent, particularly in urinary tract (33.8%), hematologic (37.4%), and endocrine gland cancers (29.4%). In patients aged over 50, multimorbidity was markedly prevalent across nearly all cancer types. The highest proportions were seen in hematologic (65.8%), respiratory (62.7%), and urinary tract cancers (61.9%). These findings highlight a clear age gradient in multimorbidity burden, with older adults showing significantly higher co-occurrence of chronic conditions, underscoring the need for age-adapted clinical care and resource planning.

### Frequency and distribution of chronic conditions and hospital mortality by age group

**Fig. 4:**
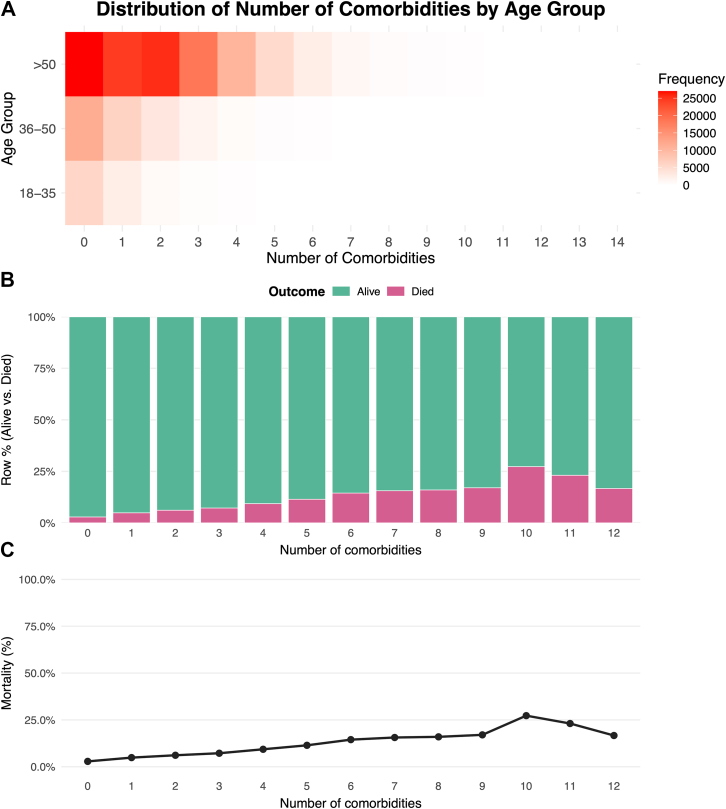
**Distribution of number of morbidities by age group and in-hospital outcome.** A) Heatmap of distribution of number of comorbidities across age groups. B) Proportion of in-hospital outcomes (alive vs. died) across comorbidity counts. C) In-hospital mortality rate (%) by number of comorbidities.

**Fig. 5:**
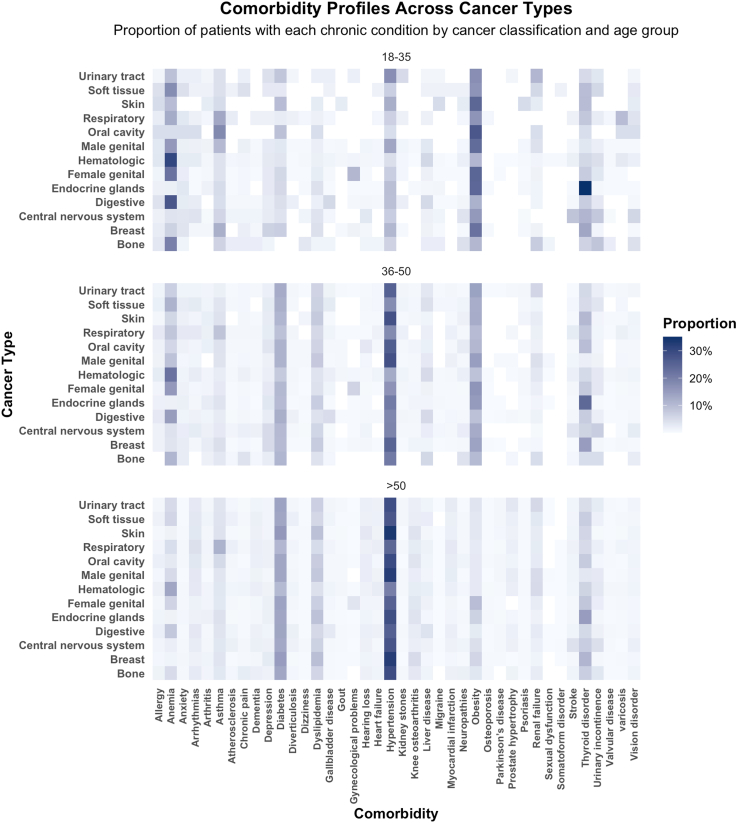
**Comorbidity profiles across cancer types**. Proportion of patients presenting with specific chronic conditions by cancer type and age group.

The distribution of comorbidities differed significantly across age groups (p < 0.001). Fig. 4 A, B and C shows this age-related gradient: most younger adults had 0-1 comorbidity, while older patients exhibited a wider and right-skewed distribution, with some individuals presenting up to 14 chronic conditions. The prevalence of specific chronic conditions varied across age groups among cancer patients (Fig. 5). In the 18–35 age group, the most common comorbidities were obesity (18.9%), anemia (14%), and thyroid disorders (9.7%). Among individuals aged 36–50, hypertension (21.5%) was the most frequent, followed by obesity (12.3%) and diabetes (10.7%). In patients over 50, hypertension remained the most prevalent comorbidity, affecting 28.1%, followed by diabetes (13.4%) and thyroid disorders (7.2%). These profiles reflects an age-related shift from nutritional and endocrine disorders in younger adults to a higher burden of cardiometabolic conditions in older populations. In the bivariate analysis, in-hospital mortality increased progressively with the number of comorbidities, from 2.9% in patients without comorbidities to over 27% in those with ≥10 comorbidities (p < 0.001). Mortality was also significantly higher in men (7.3%) compared to women (4.6%) (p < 0.001), and in adults aged ≥51 years (6.5%) compared to those aged 18–35 (2.9%) and 36–50 (3.0%) years (p < 0.001). In the multivariable analysis, a higher number of comorbidities remained independently associated with increased risk of in-hospital mortality (OR 1.26; 95% CI 1.25–1.27; p < 0.001). Female sex was associated with lower risk (OR 0.63; 95% CI 0.60–0.66; p < 0.001), while being ≥51 years was associated with higher risk compared to 18–35 years (OR 1.58; 95% CI 1.40–1.79; p < 0.001). No significant difference was observed for patients aged 36–50 years compared to the 18–35 group (OR 1.00; 95% CI 0.87–1.16; p = 0.96) (Table 1). The logistic regression model showed a Nagelkerke's R^2^ of 0.048.

**Table 1:** In-hospital mortality according to number of comorbidities, sex, and age group.

	Univariate analysis		Multivariate analysis	
	OR (95% CI)	p-value	OR (95% CI)	p-value
Number of comorbidities	1.29 (1.28–1.3)	<0.001	1.26 (1.25–1.27)	<0.001
Female (ref: male)	0.61 (0.58–0.63)	<0.001	0.63 (0.60–0.66)	<0.001
36–50 years old (ref: 18–35 years old)	1.04 (0.9–1.19)	0.623	1.00 (0.87–1.16)	0.96
≥51 years old (ref: 18–35 years old)	2.36 (2.09–2.67)	<0.001	1.58 (1.40–1.79)	<0.001

Crude in-hospital mortality rates and adjusted odds ratios (OR) with 95% confidence intervals (95% CI) from multivariable logistic regression are shown.

### Frequent multimorbidity combinations by sex and age group

**Fig. 6:**
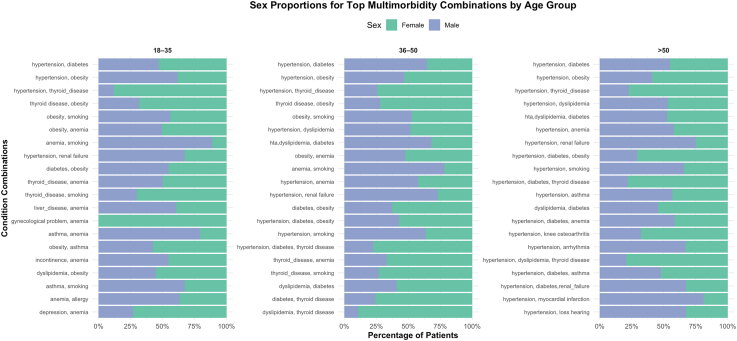
**Sex proportions for top multimorbidity combinations by age group**. Sex distribution of the most frequent multimorbidity combinations among cancer patients, stratified by age group.

The most common multimorbidity combinations differed notably by sex and age group (Fig. 6). Among females aged 18–35, the most frequent combinations were thyroid disease with obesity (11.8%), followed by gynecological problems with anemia (5.8%) and obesity with smoking (4%). In males of the same age group, the top combinations were hypertension with obesity and obesity with anemia (both 6.3%), and obesity with smoking (5.6%). For females aged 36–50, leading combinations included hypertension with obesity (4.4%), hypertension with diabetes (3.8%), and thyroid disease with obesity (2.8%). In males, the most frequent were hypertension with diabetes in both digestive (10.7%) and urinary cancers (4.5%), followed by anemia with smoking (3%). Among individuals over 50, hypertension with diabetes was the predominant combination in both sexes, affecting 13.6% of males and 8.8% of females, primarily in digestive cancers. Other frequent combinations in females included hypertension with thyroid disease (3.9%) and in males, repeated clustering of hypertension and diabetes across urinary (5.9%) and male genital cancers (5.8%).

These stratified profiles highlight the complexity and heterogeneity of multimorbidity in cancer patients across sex and age groups. Table S3 provides a comprehensive breakdown of all chronic conditions by cancer type, age, and multimorbidity status, offering granular evidence to contextualize the observed combinations and support future clinical interpretation.

## Discussion

This study provides one of the first large-scale country-level portraits of multimorbidity among hospitalized adults with cancer in Latin America, using the nationwide DRG database, we characterized age-specific multimorbidity profiles and common chronic conditions, highlighting patterns that are clinically actionable. This study analyzed over 4.7 million hospitalizations and identified 149,270 cancer patients across Chile. Nearly half of all cancer-related hospitalizations involved multimorbidity, with a strong age gradient from 14% in young adults to 55% in older patients. Obesity and anemia predominated among younger adults, while hypertension and diabetes were most frequent in older groups. In international studies on multimorbidity, large databases such as national health data and health statistics have been used,^22^ national cancer registries,^19^^,^^23^ national surveys,^24^ among others. The determination of which chronic diseases to consider assessing multimorbidity that we use corresponds to a previously published method.^7^, ^8^, ^9^ However, the heterogeneity in the definition of multimorbidity regarding which chronic diseases to include prevents these studies from being comparable with each other. This study is the first study in a Latin American population that analyzes multimorbidity in cancer patients. The findings underscore the importance of age-specific healthcare strategies to address the complex needs of cancer patients across different life stages.

Age-related differences in cancer type likely reflect distinct etiologic and care pathways. Hematologic and genital cancers were proportionally more frequent in younger adults, whereas breast cancer predominated at middle ages, and digestive and urinary tract cancers dominated in older populations. These patterns are consistent with national cancer statistics in Chile,^1^ where prostate, colorectal, and stomach cancers are leading types. The higher proportion of hematologic malignancies in younger adults may be explained by referral patterns to tertiary centers and treatment intensity in this group, while the predominance of digestive and urinary tract cancers at older ages aligns with cumulative exposure to metabolic and environmental risks.^25^, ^26^, ^27^ Together, these differences underscore the need for age-tailored prevention and screening strategies to address distinct oncologic profiles across the life course.

Multimorbidity, defined as the presence of two or more chronic conditions, shows a marked increase with age. It affects 14% of patients aged 18–35, 24.9% of those aged 36–50, and 55.5% of patients over 50. This trend reflects the accumulation of chronic health conditions over time and underscores the need for integrated care approaches for older adults. Previous studies have reported a 34% prevalence of multimorbidity among Chilean adults aged 15 and older, emphasizing the broader public health implications of this phenomenon.^28^ The prevalence of multimorbidity also varies significantly across cancer types: respiratory, urinary, digestive and hematologic cancers show the highest rates (>50%), while endocrine, bone and cartilage cancers present the lowest (<35%). The steep age gradient likely arises from accumulation of cardio-metabolic risk, age-related physiological changes, and longer exposure windows to behavioral and environmental determinants.^29^ Younger adults show a different profile, more obesity, anemia and thyroid disorders, suggesting contributions from nutritional transitions, reproductive/thyroid health, and treatment-related toxicities in intensively managed cancers. In contrast, older adults present classic hypertension/diabetes/dyslipidemia clusters, which have important implications for cardiovascular risk management during cancer care.

To contextualize our findings, we compared the prevalence of selected chronic conditions in our cohort with international estimates. Overall, the prevalence of hypertension, diabetes, COPD and stroke in our population falls within the lower-to-middle range of values reported in large cohort studies, while psychological conditions such as depression appear notably less frequent.^30^ For example, hypertension affected 21.5% of individuals aged 36–50 and 28.1% of those over 50 in our data, compared with much higher rates in the Childhood Cancer Survivor Study. Similarly, diabetes prevalence (10.7%–13.4%) was below the upper ranges reported internationally.^31^ COPD and stroke also showed values consistent with published estimates,^19^^,^^30^ while depression and anxiety appeared underrepresented, potentially reflecting underdiagnosis or underreporting in administrative data. These differences highlight both biological and contextual factors, including diagnostic practices, health system reporting, and outpatient management, that may shape the observed prevalence patterns in Chile compared to high-income countries.^30^ Additionally, priori international evidence provides context to our results. In Canada, cancer patients frequently co-occur with hypertension (8%) or osteoarthritis (6.2%), and with anxiety (25%) with marked age but not sex differences.^32^ Similar studies reported that nearly 80% of patients with urinary tract, leukemia, and lung cancers presented with multimorbidity, compared with about 60% in cervical cancer.^20^ In the UK Biobank, breast cancer patients most frequently had hypertension, asthma, thyroid disorder, and diabetes.^33^ In Spain, lung cancer patients were reported with COPD, diabetes, and heart disease.^19^

In our cohort, age-specific profiles mirrored this international evidence. Among patients over 50 years, multimorbidity was frequent, with hypertension/diabetes combinations most common, alongside clusters involving dyslipidemia and thyroid disorders. These profiles highlight the high burden of chronic conditions among older adults with cancer and reinforce the need for integrated, age-specific care strategies to manage multimorbidity in this population. Furthermore, the hypertension/obesity cluster has been associated with a higher probability of 4-year mortality (OR 1.11; CI 95% 1.02–1.21).^34^ Notably, in Chile, the co-occurrence of depression and cardiovascular disease has also been documented in cancer patients.^28^

These findings have significant implications for healthcare delivery. They highlight the necessity for personalized, age- and sex-specific interventions to manage cancer patients effectively. Integrated care models that address multiple chronic conditions simultaneously are essential, especially for older adults with a high burden of multimorbidity. Moreover, public health policies should focus on early detection and management of chronic diseases to mitigate their impact on cancer outcomes. These findings are generalizable to hospitalized adult cancer patients in Chile, as well as to countries undergoing a similar demographic transition and with a level of development comparable to that of Chile.

Some limitations of this study relate primarily to the information available in the database. Key variables for the study of multimorbidity in cancer patients, such as cancer stage or age at diagnosis, were not included. Nevertheless, the records in the Chilean DRG database have been described as accurate and complete. Prior research has identified five common approaches to validating multimorbidity studies: assessing associations with clinical outcomes, evaluating stability across subsamples and methods, examining clinical plausibility, and exploring shared determinants.^35^ Our analysis is based on clinical plausibility, as it is not possible to assess associations with long-term outcomes using hospitalization data alone. Although this study offers valuable insights, the exclusive reliance on inpatient data may underrepresent chronic conditions managed in outpatient settings. Future research should explore multimorbidity in ambulatory care and its effects on quality of life and survival among cancer patients and even perform multilevel analyses considering health system characteristics and geographic factors related to hospital mortality.

### Conclusions

Electronic health records have proven valuable for understanding multimorbidity profiles across countries, as reported in a Canadian study.^36^ The use of large-scale health databases, increasingly supported by artificial intelligence-driven analytics has been recognized as a powerful tool for informing strategies to address multimorbidity in cancer patients.^37^ This study contributes to this growing body of evidence by highlighting the complexity of cancer care in Chile, revealing distinct age- and sex-specific profiles in both cancer types and multimorbidity profiles. These findings underscore the urgent need for comprehensive, personalized, and context-sensitive healthcare strategies to improve outcomes for cancer patients across all age groups.

## Contributors

YAB and ID conceived the study. YAB conducted the data extraction and analysis, and drafted the initial manuscript. CC, CS, and RA contributed to the interpretation of results and critically revised the manuscript. MA provided methodological support in chronic disease classification and multimorbidity profiles and verified the analyses. YAB, MA and ID had full access to all the raw data used in this study. ID supervised the overall project, contributed to the final manuscript version and had final responsibility for the decision to submit the manuscript for publication. All authors reviewed and approved the final version and take responsibility for the accuracy and integrity of the data presented.

## Data sharing statement

Individual-level data used in this study are derived from a publicly available hospital discharge dataset. The database was downloaded from the Chilean National Health Fund (Fondo Nacional de Salud, FONASA) on January 29, 2025, via the following publicly accessible portal: https://public.tableau.com/views/PropuestaTableroGRD/PropuestaTableroGRD?%3AshowVizHome=no#1. All aggregated data and code used for this analysis are available in the GitHub repository: https://github.com/ybernalg/cancer_multimorbidity_chile.

## AI statement of use

The authors used OpenAI's (ChatGPT-4 model, 2024 version) to assist in improving the English grammar, clarity, and formatting of the manuscript text. The model was also used to format Markdown documentation in the project's GitHub repository. No AI tools were employed for data analysis, statistical interpretation, or content generation related to the study results. All text revisions suggested by AI were reviewed and approved by the authors.

## Declaration of interests

All authors declare no competing interests.

## References

[1] Bray F., Laversanne M., Sung H. (2024). Global cancer statistics 2022: GLOBOCAN estimates of incidence and mortality worldwide for 36 cancers in 185 countries. CA Cancer J Clin.

[2] Piñeros M., Laversanne M., Barrios E. (2022). An updated profile of the cancer burden, patterns and trends in Latin America and the Caribbean. Lancet Reg Health Am.

[3] Camargo M.C., Feliu A., Stern M.C., Villarreal-Garza C., Ferreccio C., Espina C. (2023). The Latin America and the Caribbean code against cancer: an opportunity for empowerment and progress. Lancet Reg Health Am.

[4] Zullig L.L., Sung A.D., Khouri M.G. (2022). Cardiometabolic comorbidities in cancer survivors: jacc: cardiooncology state-of-the-art review. J Am Coll Cardiol.

[5] Reste J.Y.L., Nabbe P., Manceau B. (2013). The European general practice research network presents a comprehensive definition of multimorbidity in family medicine and long term care, following a systematic review of relevant literature. J Am Med Dir Assoc.

[6] Alvarez-Galvez J., Vegas-Lozano E. (2022). Discovery and classification of complex multimorbidity patterns: unravelling chronicity networks and their social profiles. Sci Rep.

[7] Koller D., Schön G., Schäfer I., Glaeske G., van den Bussche H., Hansen H. (2014). Multimorbidity and long-term care dependency—a five-year follow-up. BMC Geriatr.

[8] Schang L., Koller D., Franke S., Sundmacher L. (2019). Exploring the role of hospitals and office-based physicians in timely provision of statins following acute myocardial infarction: a secondary analysis of a nationwide cohort using cross-classified multilevel models. BMJ Open.

[9] van den Bussche H., Koller D., Kolonko T. (2011). Which chronic diseases and disease combinations are specific to multimorbidity in the elderly? Results of a claims data based cross-sectional study in Germany. BMC Public Health.

[10] Sarmiento L.L. (2022). Patrones de multimorbilidad en la población chilena mayor de 15 años: análisis de datos epidemiológicos de la encuesta nacional de salud 2016-2017. Revista Médica Clínica Las Condes.

[11] Nazar G., Díaz-Toro F., Petermann-Rocha F. (2023). Multimorbidity and 11-year mortality in adults: a prospective analysis using the Chilean national health survey. Health Promot Int.

[12] Souza D.L.B., Oliveras-Fabregas A., Minobes-Molina E., de Camargo Cancela M., Galbany-Estragués P., Jerez-Roig J. (2021). Trends of multimorbidity in 15 European countries: a population-based study in community-dwelling adults aged 50 and over. BMC Public Health.

[13] Makovski T.T., Schmitz S., Zeegers M.P., Stranges S., van den Akker M. (2019). Multimorbidity and quality of life: systematic literature review and meta-analysis. Ageing Res Rev.

[14] Ritchie C.S., Kyale E., Fisch M.J. (2011). Multimorbidity: an issue of growing importance for oncologists. JCO Oncology Practice.

[15] Keats M.R., Cui Y., DeClercq V., Grandy S.A., Sweeney E., Dummer T.J.B. (2021). Burden of multimorbidity and polypharmacy among cancer survivors: a population-based nested case–control study. Support Care Cancer.

[16] Jiang C., Deng L., Karr M.A. (2022). Chronic comorbid conditions among adult cancer survivors in the United States: results from the national health interview survey, 2002-2018. Cancer.

[17] Macinko J., Andrade F.C.D., Nunes B.P., Guanais F.C. (2019). Primary care and multimorbidity in six Latin American and Caribbean countries. Rev Panam Salud Publica.

[18] Luque-Fernandez M.A., Gonçalves K., Salamanca-Fernández E. (2020). Multimorbidity and short-term overall mortality among colorectal cancer patients in Spain: a population-based cohort study. Eur J Cancer.

[19] Niksic M., Redondo-Sanchez D., Chang Y.-L. (2021). The role of multimorbidity in short-term mortality of lung cancer patients in Spain: a population-based cohort study. BMC Cancer.

[20] Koné A.P., Scharf D. (2021). Prevalence of multimorbidity in adults with cancer, and associated health service utilization in Ontario, Canada: a population-based retrospective cohort study. BMC Cancer.

[21] Harrison C., Fortin M., van den Akker M. (2021). Comorbidity versus multimorbidity: why it matters. J Comorb Multimorb.

[22] Zheng D.D., Loewenstein D.A., Christ S.L. (2021). Multimorbidity patterns and their relationship to mortality in the US older adult population. PLoS One.

[23] Fowler H., Belot A., Ellis L. (2020). Comorbidity prevalence among cancer patients: a population-based cohort study of four cancers. BMC Cancer.

[24] Yamashita H., Takahashi Y., Ishizaki T., Imura H., Nakayama T. (2020). Associations of multimorbidity with breast, cervical, and colorectal cancer screening delivery: a cross-sectional study of a nationally representative Japanese sample. Cancer Epidemiology.

[25] Préndez M., Nova P., Romero H., Mendes F., Fuentealba R. (2023). Representativeness of the particulate matter pollution assessed by an official monitoring station of air quality in Santiago, Chile: projection to human health. Environ Geochem Health.

[26] Muñoz-Quezada M.T., Iglesias V., Zúñiga-Venegas L. (2025). Exposure to pesticides in Chile and its relationship with carcinogenic potential: a review. Front Public Health.

[27] Steinmaus C.M., Ferreccio C., Romo J.A. (2013). Drinking water arsenic in northern Chile: high cancer risks 40 years after exposure cessation. Cancer Epidemiol Biomarkers Prev.

[28] Nazar G., Díaz-Toro F., Concha-Cisternas Y. (2023). Latent class analyses of multimorbidity and all-cause mortality: a prospective study in Chilean adults. PLoS One.

[29] Shi J., Liu C., Zheng X. (2025). Novel metabolic prognostic score for predicting survival in patients with cancer. Sci Rep.

[30] Ahmad T.A., Gopal D.P., Chelala C., Dayem Ullah A.Z., Taylor S.J. (2023). Multimorbidity in people living with and beyond cancer: a scoping review. Am J Cancer Res.

[31] Gibson T.M., Li Z., Green D.M. (2017). Blood pressure status in adult survivors of childhood cancer: a report from the St. Jude lifetime cohort Study. Cancer Epidemiol Biomarkers Prev.

[32] Koné A.P., Scharf D., Tan A. (2023). Multimorbidity and complexity among patients with cancer in Ontario: a retrospective cohort study exploring the clustering of 17 chronic conditions with cancer. Cancer Control.

[33] Foster M., Niedzwiedz C.L. (2021). Associations between multimorbidity and depression among breast cancer survivors within the UK biobank cohort: a cross-sectional study. BMC Cancer.

[34] Ioakeim-Skoufa I., González-Rubio F., Aza-Pascual-Salcedo M. (2024). Multimorbidity patterns and trajectories in young and middle-aged adults: a large-scale population-based cohort study. Front Public Health.

[35] Dhafari T.B., Pate A., Azadbakht N. (2024). A scoping review finds a growing trend in studies validating multimorbidity patterns and identifies five broad types of validation methods. J Clin Epidemiol.

[36] Nicholson K., Bauer M., Terry A.L., Fortin M., Williamson T., Thind A. (2017). The multimorbidity cluster analysis tool: identifying combinations and permutations of multiple chronic diseases using a record-level computational analysis. BMJ Health Care Inform.

[37] Cesario A., D'Oria M., Calvani R. (2021). The role of artificial intelligence in managing multimorbidity and cancer. J Personalized Med.

