# Supplementary material for: Multimorbidity profile among cancer-related hospitalization events in younger and older patients: a large-scale nationwide cross-sectional study
Source: Lancet Reg Health Am. 2025 Nov 25;53:101308. doi: 10.1016/j.lana.2025.101308 (PMC12685555; doi:10.1016/j.lana.2025.101308)
Supplement: Supplementary Tables [file mmc1.docx]

**Supplementary tables**

**Table S1. ICD-10 codes used to define each chronic condition**. This table outlines the classification of chronic diseases included in the multimorbidity analysis and their corresponding diagnostic groupings and ICD-10 codes.

| **Diagnostic Group** | **ICD Codes** |
| --- | --- |
| High Blood Pressure | I10, I11, I12, I13, I14, I15 |
| Myocardial Infarction | I20, I21, I25 |
| Other Heart Diseases | I30-I52 |
| Varicose Veins | I83, I87 |
| Osteoarthritis | M15-M19 |
| Osteoporosis | M80-M82 |
| Arthritis | M05, M06, M79 |
| Chronic Pain | M40-M54 |
| Chronic Allergy | J30, L23-L29, K27-K29 |
| Asthma | J45 |
| Chronic Bronchitis, Emphysema, COPD | J40-J44 |
| Diabetes | E10-E14 |
| Stomach or Duodenal Ulcer | K25, K26 |
| Urinary Incontinence or Urine Control Problems | N39 |
| High Cholesterol | E78 |
| Vision Problems | H17-H54 |
| Chronic Skin Problems | L20-L40 |
| Liver Diseases | K70-K76 |
| Mental Health Conditions | F32, F33, F41, F99 |
| Stroke | I60-I69, G45 |
| Migraine or Frequent Headache | G43, R51 |
| Haemorrhoids | I84 |
| Thyroid Problems | E00-E07 |
| Kidney Problems | N00-N20 |
| Prostate Problems (Men Only) | N40-N42 |
| Menopausal Problems (Women Only) | N95 |
| Permanent Injuries or Defects Caused by an Accident | T90-T98 |
| Obesity | E66 |
| Loss of Hearing | H90, H91 |
| Gallbladder Diseases | K80, K81 |
| Atherosclerosis | I65-I73 |
| Diverticulosis | K57 |
| Neuropathies | G50-G64 |
| Dizziness | H81, H82, R42 |
| Dementia | F00-F05, G30-G31, R54 |
| Urinary Incontinence | N39, R32 |
| Anemia | D50-D64 |
| Sexual Disorders | F52, N48 |
| Insomnia | G47, F51 |
| Tobacco Use Disorders | F17 |
| Gout | E79, M10 |

**Table S2. Sociodemographic and clinical characteristics of cancer patients by age group.** The table includes the distribution of sex, year of discharge, nationality, type of discharge, and self-reported ethnicity among hospitalized cancer patients aged 18–35, 36–50, and >50 years.

| **Variable** | **18–35 years old (n=9,629)** | **36–50 years old (n=24,031)** | **>50 years old (n=115,610)** | **p-value** |
| --- | --- | --- | --- | --- |
| **Age (years old)** |  |  |  | <0.001 |
| Mean (SD) | 28.7 (4.79) | 44.8 (4.52) | 68.4 (9.52) |  |
| Median [p25-p75] | 30.0 [25, 33] | 45.0 [41, 49] | 68.0 [61, 78] |  |
| **Sex** |  |  |  | <0.001 |
| Male | 3846 (39.9%) | 6609 (27.5%) | 53551 (46.3%) |  |
| Female | 5782 (60.1%) | 17422 (72.5%) | 62059 (53.7%) |  |
| **Year of Discharge** |  |  |  | <0.001 |
| 2019 | 2006 (20.8%) | 5100 (21.2%) | 25891 (22.4%) |  |
| 2020 | 1622 (16.8%) | 3857 (16.1%) | 18942 (16.4%) |  |
| 2021 | 2007 (20.8%) | 4774 (19.9%) | 21756 (18.8%) |  |
| 2022 | 1916 (19.9%) | 5004 (20.8%) | 23207 (20.1%) |  |
| 2023 | 2078 (21.6%) | 5296 (22.0%) | 25814 (22.3%) |  |
| **Nationality** |  |  |  | <0.001 |
| Chilean | 8900 (92.4%) | 22715 (94.5%) | 113603 (98.3%) |  |
| Non-Chilean | 728 (7.6%) | 1316 (5.5%) | 2007 (1.7%) |  |
| **Discharge Type** |  |  |  | <0.001 |
| Home discharge or home hospitalization | 8937 (92.8%) | 22696 (94.4%) | 105008 (90.8%) |  |
| Referral to other healthcare facilities | 318 (3.3%) | 421 (1.8%) | 232 (2%) |  |
| Death | 278 (2.9%) | 718 (3%) | 7567 (6.5%) |  |
| Voluntary discharge | 72 (0.7%) | 163 (0.7%) | 660 (0.6%) |  |
| Unauthorized discharge | 23 (0.2%) | 33 (0.1%) | 55 (0.1%) |  |
| **Self-reported Ethnicity** |  |  |  | <0.001 |
| Chilean Indigenous Peoples | 9449 (98.1%) | 23663 (98.5%) | 114028 (98.6%) |  |
| None, or other | 180 (1.9%) | 368 (1.5%) | 1581 (1.4%) |  |

**Table S3. Distribution of cancer types and chronic conditions by age group and multimorbidity status.** The table shows the frequency and percentage of each cancer diagnosis and comorbid condition stratified by age category (18–35, 36–50, and >50 years) and multimorbidity status (with or without multimorbidity).

|  | **18-35 years old** | | **36-50 years old** | | **>50 years old** | |
| --- | --- | --- | --- | --- | --- | --- |
|  | **Witout multimorbidity (n =8,275)** | **With multirmorbidity (n= 1,353)** | **Witout multimorbidity (n= 18,051)** | **With multirmorbidity (n= 5,980)** | **Witout multimorbidity (n= 51,414)** | **With multirmorbidity (n= 64,196)** |
| **Cancer type** | | | | | | |
| Soft tissue | 138 (1.7%) | 17 (1.2%) | 184 (1%) | 77 (1.3%) | 515 (1%) | 655 (1%) |
| Digestive | 671 (8.1%) | 128 (9.2%) | 3391 (18.8%) | 1176 (19.7%) | 17725 (34.5%) | 23486 (36.6%) |
| Female genital | 1,424 (17.2%) | 230 (16.5%) | 2668 (14.8%) | 1201 (20.1%) | 3055 (5.9%) | 5155 (8%) |
| Male genital | 1,511 (18.3%) | 104 (7.4%) | 824 (4.6%) | 118 (2%) | 4075 (7.9%) | 3837 (6%) |
| Endocrine glands | 1,341 (16.2%) | 257 (19.1%) | 1949 (10.8%) | 812 (13.6%) | 1521 (3%) | 1711 (2.7%) |
| Hematologic | 1,159 (14.1%) | 358 (25.6%) | 1124 (6.2%) | 670 (11.2%) | 2813 (5.5%) | 5418 (8.4%) |
| Breast | 940 (11.4%) | 69 (8.2%) | 5414 (30%) | 1024 (17.1%) | 9699 (18.9%) | 7590 (11.8%) |
| Oral cavity | 75 (0.9%) | 6 (0.4%) | 228 (1.3%) | 68 (1.1%) | 961 (1.9%) | 851 (1.3%) |
| Bone | 135 (1.6%) | 24 (1.7%) | 129 (0.7%) | 36 (0.6%) | 239 (0.5%) | 228 (0.4%) |
| Skin | 141 (1.7%) | 14 (1%) | 479 (2.7%) | 89 (1.5%) | 3063 (6%) | 2873 (4.5%) |
| Respiratory | 143 (1.7%) | 40 (2.9%) | 426 (2.4%) | 151 (2.5%) | 2850 (5.5%) | 4784 (7.5%) |
| Central nervous system | 447 (5.4%) | 74 (5.3%) | 478 (2.6%) | 171 (2.9%) | 828 (1.6%) | 990 (1.5%) |
| Urinary tract | 150 (1.8%) | 32 (2.3%) | 757 (4.2%) | 387 (6.5%) | 4070 (7.9%) | 6618 (10.3%) |
| **Chronic diseases** | | | | | | |
| High Blood Pressure | 120 (1.5%) | 292 (21.7%) | 1156 (6.4%) | 2908 (48.6%) | 10058 (19.6%) | 49946 (77.8%) |
| Dyslipidemia | 27 (0.3%) | 104 (7.7%) | 161 (0.9%) | 939 (15.7%) | 656 (1.3%) | 13512 (21%) |
| Chronic Pain | 18 (0.2%) | 34 (2.5%) | 46 (0.3%) | 112 (1.9%) | 108 (0.2%) | 975 (1.5%) |
| Vision Problems | 23 (0.3%) | 34 (2.5%) | 26 (0.1%) | 93 (1.6%) | 120 (0.2%) | 1875 (2.9%) |
| Knee Osteoarthritis | 1 (0.0%) | 4 (0.3%) | 29 (0.2%) | 117 (2.0%) | 420 (0.8%) | 5690 (8.9%) |
| Diabetes | 57 (0.7%) | 178 (13.2%) | 337 (1.9%) | 1803 (30.2%) | 1765 (3.4%) | 28012 (43.6%) |
| Myocardial Infarction | 0 (0%) | 4 (0.3%) | 17 (0.1%) | 86 (1.4%) | 144 (0.3%) | 3873 (6%) |
| Thyroid Disorders | 330 (4%) | 312 (23.1%) | 773 (4.3%) | 1373 (23.0%) | 1569 (3.1%) | 12581 (19.6%) |
| Arrhythmias | 28 (0.3%) | 45 (3.3%) | 56 (0.3%) | 183 (3.1%) | 356 (0.7%) | 5456 (8.5%) |
| Obesity | 422 (5.1%) | 532 (39.3%) | 759 (4.2%) | 1857 (31.1%) | 704 (1.4%) | 7830 (12.2%) |
| Gout | 5 (0.1%) | 6 (0.4%) | 5 (0.0%) | 23 (0.4%) | 33 (0.1%) | 601 (0.9%) |
| Prostate Hyperplasia | 0 (0%) | 2 (0.1%) | 7 (0.0%) | 22 (0.4%) | 369 (0.7%) | 2942 (4.6%) |
| Varicose Veins | 26 (0.3%) | 27 (2.0%) | 24 (0.1%) | 64 (1.1%) | 70 (0.1%) | 757 (1.2%) |
| Liver Disease | 34 (0.4%) | 107 (7.9%) | 122 (0.7%) | 411 (6.9%) | 464 (0.9%) | 4371 (6.8%) |
| Depression | 53 (0.6%) | 96 (7.1%) | 164 (0.9%) | 450 (7.5%) | 292 (0.6%) | 2767 (4.3%) |
| Asthma | 139 (1.7%) | 151 (11.2%) | 200 (1.1%) | 457 (7.6%) | 861 (1.7%) | 8212 (12.8%) |
| Gynecologic Problems | 59 (0.7%) | 62 (4.6%) | 71 (0.4%) | 227 (3.8%) | 106 (0.2%) | 691 (1.1%) |
| Atherosclerosis | 6 (0.1%) | 16 (1.2%) | 14 (0.1%) | 39 (0.7%) | 81 (0.2%) | 1936 (3.0%) |
| Osteoporosis | 0 (0%) | 3 (0.2%) | 6 (0.0%) | 9 (0.2%) | 26 (0.1%) | 420 (0.7%) |
| Renal Failure | 23 (0.3%) | 64 (4.7%) | 55 (0.3%) | 332 (5.6%) | 250 (0.5%) | 6354 (9.9%) |
| Stroke | 17 (0.2%) | 37 (2.7%) | 27 (0.1%) | 115 (1.9%) | 111 (0.2%) | 2140 (3.3%) |
| Heart Failure | 5 (0.1%) | 13 (1.0%) | 11 (0.1%) | 68 (1.1%) | 46 (0.1%) | 3199 (5.0%) |
| Hearing Loss | 7 (0.1%) | 23 (1.7%) | 30 (0.2%) | 70 (1.2%) | 318 (0.6%) | 3153 (4.9%) |
| Gallbladder Disease | 14 (0.2%) | 40 (3.0%) | 99 (0.5%) | 233 (3.9%) | 381 (0.7%) | 2892 (4.5%) |
| Somatic Symptoms | 0 (0%) | 4 (0.3%) | 0 (0%) | 0 (0%) | 1 (0.0%) | 16 (0.0%) |
| Diverticulosis | 3 (0.0%) | 13 (1.0%) | 25 (0.1%) | 97 (1.6%) | 151 (0.3%) | 2267 (3.5%) |
| Arthritis | 16 (0.2%) | 21 (1.6%) | 92 (0.5%) | 207 (3.5%) | 186 (0.4%) | 1428 (2.2%) |
| Valvular Disease | 3 (0.0%) | 9 (0.7%) | 5 (0.0%) | 25 (0.4%) | 19 (0.0%) | 755 (1.2%) |
| Neuropathies | 14 (0.2%) | 34 (2.5%) | 24 (0.1%) | 106 (1.8%) | 66 (0.1%) | 718 (1.1%) |
| Dizziness | 7 (0.1%) | 11 (0.8%) | 9 (0.0%) | 24 (0.4%) | 33 (0.1%) | 321 (0.5%) |
| Dementia | 5 (0.1%) | 15 (1.1%) | 10 (0.1%) | 47 (0.8%) | 222 (0.4%) | 2743 (4.3%) |
| Urinary Incontinence | 56 (0.7%) | 100 (7.4%) | 76 (0.4%) | 316 (5.3%) | 339 (0.7%) | 3491 (5.4%) |
| Kidney Stones | 16 (0.2%) | 23 (1.7%) | 25 (0.1%) | 104 (1.7%) | 52 (0.1%) | 714 (1.1%) |
| Anemia | 473 (5.7%) | 440 (32.5%) | 809 (4.5%) | 1373 (23.0%) | 1897 (3.7%) | 11843 (18.4%) |
| Anxiety | 51 (0.6%) | 81 (6.0%) | 91 (0.5%) | 234 (3.9%) | 95 (0.2%) | 657 (1.0%) |
| Psoriasis or Chronic Skin Disease | 10 (0.1%) | 7 (0.5%) | 18 (0.1%) | 51 (0.9%) | 51 (0.1%) | 453 (0.7%) |
| Migraine | 13 (0.2%) | 11 (0.8%) | 13 (0.1%) | 40 (0.7%) | 19 (0.0%) | 79 (0.1%) |
| Parkinson’s Disease | 0 (0%) | 2 (0.1%) | 1 (0.0%) | 8 (0.1%) | 84 (0.2%) | 819 (1.3%) |
| Chronic Allergy | 42 (0.5%) | 80 (5.9%) | 107 (0.6%) | 238 (4.0%) | 270 (0.5%) | 2296 (3.6%) |
| Sexual Disorders | 2 (0.0%) | 3 (0.2%) | 0 (0%) | 5 (0.1%) | 7 (0.0%) | 35 (0.1%) |
| Insomnia | 6 (0.1%) | 23 (1.7%) | 17 (0.1%) | 69 (1.2%) | 34 (0.1%) | 563 (0.9%) |
| Tobacco Use Disorder | 353 (4.3%) | 252 (18.7%) | 763 (4.2%) | 989 (16.5%) | 1572 (3.1%) | 5830 (9.1%) |
